# HIV Risk Perception among HIV Negative or Status-Unknown Men Who Have Sex with Men in China

**DOI:** 10.1155/2014/232451

**Published:** 2014-03-30

**Authors:** Wensheng Fan, Lu Yin, Han-Zhu Qian, Dongliang Li, Yiming Shao, Sten H. Vermund, Yuhua Ruan, Zheng Zhang

**Affiliations:** ^1^Department of Public Health, College of Health and Human Services, Western Kentucky University, Bowling Green, KY 42101, USA; ^2^Vanderbilt Institute for Global Health, Vanderbilt University School of Medicine, Nashville, TN 37203, USA; ^3^Department of Medicine, Vanderbilt University School of Medicine, Nashville, TN 37203, USA; ^4^Chaoyang Center for Disease Control and Prevention, 25 Huaweili, Panjiayuan, Chaoyang District, Beijing 100021, China; ^5^State Key Laboratory for Infectious Disease Prevention and Control, National Center for AIDS/STD Control and Prevention, Chinese Center for Disease Control and Prevention, Collaborative Innovation Center for Diagnosis and Treatment of Infectious Diseases, 155 Changbai Road, Changping District, Beijing 102206, China; ^6^Department of Pediatrics, Vanderbilt University School of Medicine, Nashville, TN 37203, USA

## Abstract

*Objective.* To evaluate HIV risk perception and its associated factors among Chinese MSM. *Methods.* A cross-sectional study was conducted among MSM with an HIV negative or unknown status in Beijing, China, between 2011 and 2012. A questionnaire interview was conducted and a blood sample was collected for HIV and syphilis testing. *Results.* Of 887 MSM who reported they were HIV negative or did not know their HIV status before recruitment, only 7.3% reported a high risk of HIV infection, 28.0% medium risk, 52.2% low risk, and 12.5% no risk. In multivariate logistic regression models using those who reported a medium self-perceived risk as a reference group, self-reported high risk of HIV perception was associated with minority ethnicity (odds ratio [OR]: 2.91; 95% confidence interval [CI]: 1.03–8.19), self-reported history of sexually transmitted diseases (OR: 2.27; 95% CI: 1.25–4.10), and HIV testing times since the last HIV testing (OR: 0.47; 95% CI: 0.26–0.84); low self-perceived risk of HIV infection was related to full-time employment (OR: 1.58; 95% CI: 1.15–2.18) and illicit drug use (OR: 0.28; 95% CI: 0.10–0.75). *Conclusions.* The HIV/AIDS epidemic is rapidly rising among Beijing MSM, but more than half MSM did not perceive this risk.

## 1. Introduction

Data from Europe, North America, Latin America, Asia, and Sub-Saharan Africa indicate that human immunodeficiency virus/acquired immunodeficiency syndrome (HIV/AIDS) has been increasing over time, especially among men who have sex with men (MSM) [1–5]. In China, 17.4% were infected through homosexual contacts in 2011, rising from 11.0% in 2007 to 7.3% in 2005 [6]. Three prospective cohort studies which had been separately conducted among MSM in Beijing during 2006–2010 and followed for one year with more than 86.0% retention reported 2.6, 3.4, and 8.1 per 100 person-years of HIV incidence rates in 2007, 2009, and 2010, respectively [7–9]. Four cross-sectional studies showed that HIV prevalence rate among MSM in Beijing significantly increased to 6.3% in 2009, from 5.8% in 2006, 4.6% in 2005, and 0.4% in 2004, representing a 15-times increase within 5 years from 2004 to 2009 [10, 11]. The rapid increase of HIV epidemic among MSM has reminded the Chinese government and researchers that comprehensive biomedical and behavioral interventions and other policies are urgently needed for preventing the spread of HIV in this high risk population.

Age, unmarried status, education, multiple or temporary sexual partners, and inconsistent condom use have been reported to be related to HIV risk among MSM [7, 11, 12]. Since China has adopted an open-door policy since the early 1980s, the Chinese society has become more tolerant towards sex including homosexuality. Lack of sex knowledge and safer sex awareness among young people [13] and pursuit of sexual pleasure without condom use among MSM are prevalent, even though various policies, strategies, and interventions for HIV/AIDS prevention and control have been introduced and applied to the MSM population [7–10]. In addition, unprotected sex often occurs under drug or alcohol use among a proportion of MSM [14–16]. Therefore, risk awareness education is especially important to prevent and control HIV transmission among MSM [17, 18].

A qualitative study among MSM in Beijing and Chongqing found that the majority of participants were aware of the high HIV epidemic among MSM, but they did not think themselves at a high risk of HIV infection [19]. We conducted a quantitative assessment of HIV risk perception and its associated factors among Chinese MSM.

## 2. Methods

### 2.1. Study Design and Population

This study was conducted among MSM in Beijing, China, from January 2010 to July 2011. The study design and study population were described in detail elsewhere [20]. In brief, a local gay volunteer grassroots organization—Chaoyang Chinese AIDS Volunteer Group—recruited MSM participants from the community, sexually transmitted disease (STD) clinics, and voluntary HIV counseling and testing clinics using various approaches, such as website advertisements, outreach to MSM-frequented venues (e.g., MSM clubs, bars, and bathhouses), and peer-referrals. The primary aim of this study was to investigate HPV prevalence using genital and anal swab specimens among HIV-infected and uninfected MSM. This paper aimed to estimate a self-perceived risk of HIV infection among HIV seronegative or status-unknown MSM. Hence, those who have known their positive status are excluded from these analyses (*N* = 233). Written informed consent was obtained before the questionnaire interview and blood collection. This study was reviewed and monitored by the Institutional Review Boards of the National Center for AIDS/STD Control and Prevention (NCAIDS) of the Chinese Center for Disease Control and Prevention (NCAIDS IRB FWA00002958) and Vanderbilt University School of Medicine (VU IRB FWA00005756).

### 2.2. Data Collection

Questionnaire data of sociodemographic factors, drug and alcohol use, preferred sexual position during anal sex, self-reported sexual orientation, multiple concurrent male sexual partners in the last 12 months, traded sex for money in the past 12 months, forced to have sex with any male partners in the past 12 months, self-reported history of sexually transmitted diseases, ever having sex with female sexual partner(s), history of HIV testing, and self-perception of HIV/AIDS risk were collected by well-trained interviewers.

### 2.3. Blood Collection and Laboratory Tests

A physical examination was conducted by trained and experienced physicians to collect circumcision status. A blood sample was collected to test for HIV and syphilis infections according to Chinese National Testing Protocols at the Institute of STD/AIDS Prevention and Treatment, Xicheng District Center for Disease Control and Prevention and the Beijing Jingcheng Venereal Hospital, Beijing, China. Each participant was assigned a unique code to link the anonymous questionnaire and blood. HIV infection status was determined by an enzyme immunoassay (Wantai Biological Medicine Company, Beijing, China), and positive samples were confirmed by HIV-uninfected 1/2 Western blot assay (HIV Blot 2.2 WB; Genelabs Diagnostics, Singapore, Singapore). Syphilis serology was determined through rapid plasma reagin (Shanghai Kehua Biotechnology Co., Ltd., Shanghai, China) and confirmed by the Treponema palladium particle assay (Fujirebio Inc., Tokyo, Japan).

### 2.4. Statistical Analysis

Data for questionnaire responses, physical examinations, and laboratory tests were entered independently by 2 study staff and verified with EpiData software (EpiData 3.1 for Windows; The EpiData Association Odense, Odense, Denmark). Completed databases were then analyzed with Statistical Analysis System (SAS 9.3 for Windows; SAS Institute Inc., Cary, NC, USA) software. Participants were asked a question: “How large do you think the risk of being infected with HIV?” And they could respond with one of four answers: (1) high risk; (2) medium risk; (3) low risk; and (4) no risk. Univariate logistic regression analyses were used to estimate the odds ratios (OR) and 95% confidence interval (CI) for the association between HIV risk perception and some demographic and behavioral factors, using medium risk as reference: (1) high risk versus medium risk; (2) low risk versus medium risk; and (3) no risk versus medium risk. Multivariable logistic regression was used to determine predictors of HIV risk perception using medium risk as the reference. All variables with *P* < 0.10 in univariate analyses were entered into the multivariate logistic model using stepwise selection. Separate logistic regression models were used to further evaluate the associations of HIV/syphilis infection with self-perceived risk of HIV infection without and with adjustment for potential confounders, such as age, ethnic, years of education, and full-time employment.

## 3. Results

A total of 1155 potential participants were recruited into the study and provided informed consent; 251 were excluded due to HIV-seropositive or unconfirmed HIV status; 17 were further excluded due to missing information on HIV risk perception. Therefore, a total of 887 HIV-uninfected participants were included in this analysis on HIV risk perception.

Table 1 presents basic characteristics of MSM who participated in this study. The average age at interview was 30.2 years; 94.0% were Han ethnics; 72.9% were never married; 52.5% received college education; 63.9% were employed full time; 2.8% ever having used illicit drugs; 24.8% reported alcohol drinking daily in the past 4 weeks. About two-thirds (65.7%) reported homosexual orientation and 33.1% reported bisexual orientation. As for the role of anal sex, 29.1% preferred receptive, 45.6% preferred insertive, and 21.8% reported dual. Among this study population, only 3.4% had never had HIV testing and 83.4% reported having HIV testing in the past 12 months. Of 887 participants, 5.3% were confirmed as HIV positive and 16.7% as syphilis positive.

Only 7.3% perceived themselves at high risk of HIV infection; 28.0% perceived themselves to be at medium risk; 52.2% at low risk; and 12.5% at no risk. In unadjusted analyses, the following factors were associated with high risk perception compared with medium risk perception: younger age (OR, 0.96; 95% CI, 0.92–1.00; *P* = 0.07) and minority ethnicity (OR, 2.87; 95% CI, 1.05–7.87; *P* = 0.04), history of sexually transmitted diseases (OR, 2.17; 95% CI, 1.22–3.88; *P* = 0.01), and ≥3 months since last HIV testing (OR, 0.48; 95% CI, 0.27–0.84; *P* = 0.01). Factors associated with low risk perception were higher education level (OR, 1.37; 95% CI, 1.01–1.87; *P* = 0.05), full-time employment (OR, 1.64; 95% CI, 1.19–2.25; *P* < 0.01), and no history of illicit drug use (OR, 0.26; 95% CI, 0.10–0.69; *P* = 0.01). Factors associated with no self-perceived risk were lower education level (OR, 0.58; 95% CI, 0.37–0.92; *P* = 0.02), bisexual orientation (OR, 1.79; 95% CI, 1.11–2.88; *P* = 0.02), and ever had sex with female sexual partner(s) (OR, 2.02; 95% CI, 1.28–3.17; *P* < 0.01) (Table 2).

The factors significant in the univariate analyses were included in multivariate analyses (Table 3). Compared with those with medium risk perception, those with high risk perception tended to be minority ethnicity (OR, 2.91; 95% CI, 1.03–8.19; *P* = 0.04), have a history of sexually transmitted diseases (OR, 2.27; 95% CI, 1.25–4.10; *P* = 0.01), and have more than 3 months since last HIV testing (OR, 0.47; 95% CI, 0.26–0.84; *P* = 0.01). Those with low risk perception tended to be a full-time employee (OR, 1.58; 95% CI, 1.15–2.18; *P* = 0.01) and have a history of using illicit drugs (OR, 0.28; 95% CI, 0.10–0.75; *P* = 0.01). Those with no perceived HIV risk were more likely to have female sexual partners (OR, 1.93; 95% CI, 1.22–3.05; *P* = 0.01).

Table 4 presents the association between HIV risk perception and HIV and syphilis infections. A U-shape association between HIV seropositivity and self-perceived HIV risk was found, and a lower prevalence of HIV infection was observed among those perceiving themselves at low risk than that among those reporting high HIV risk (3.7% versus 10.8%; OR, 0.35; 95% CI, 0.14–0.90; *P* = 0.03) after adjusting for age, ethnicity, years of education, and full-time employment. There is a marginally statistically significant association between syphilis seropositivity and self-perceived risk of HIV infection (low risk versus high risk: 13.8% versus 21.5%; OR, 0.53; 95% CI, 0.27–1.02; *P* = 0.06).

## 4. Discussions

Our study found that a very small proportion of MSM in Beijing had high risk perception and about two-thirds perceived themselves at low or no risk. Several previous studies in the USA [18, 21–23] and Netherlands [17] also reported that over half of MSM perceived that they had low or no chance of contracting HIV. However, HIV has been endemic among MSM in Beijing [10, 11, 24]. Our study showed that men with high risk perception did have a higher HIV prevalence (10.8%) than those reporting medium or low risk (6.8% and 3.7%, resp.). A marginally significant U-shape trend also was observed for syphilis seropositivity. Such associations between HIV/syphilis infection and self-perceived risk of HIV infection reminded us that it is urgent to improve health education/interventions and increase their high risk of HIV infection among this population.

Behavioral change theories suggest that self-perception plays an important role in health behavior [25]. Among respondents in our survey, 3.4% had never been tested for HIV and over two- thirds had a test within the past 6 months. There are various reasons for not taking a test among Chinese MSM, such as stigma and fear about learning their HIV status [26–28]. However, the prevalence of HIV testing was high in our study, and the coverage of HIV testing among MSM in Beijing has been expanded in the past two years [29]. Noninfected MSM tend not to take a test because they have low risk perception [23, 26, 28]. Hence, it is suggested that MSM or other high risk populations take HIV testing regularly, especially for those frequently engaging in high risk behaviors [30]. Those perceiving themselves as having a high risk of contracting HIV were more likely to seek HIV testing in the past 3 months, compared to those with perceived medium-, low-, or no risk. Providing routine HIV testing in medical care settings may increase HIV risk perception among MSM [22].

Studies have shown that those engaging in risk behaviors were likely to consider themselves at high risk [26]. In our study, it is interesting to find that a higher proportion of those perceiving themselves at high risk of HIV infection reported engaging in high risk behaviors, for example, 36.9% of those reporting unprotected anal sex and 12.3% of those reporting multiple concurrent male sexual partners. However, a moderate number of MSM still believed their chance of contracting HIV was low despite their involvement in risky sex in current and previous studies [18, 21, 22]. Though HIV testing has increased among MSM in Beijing, unprotected anal sex is still common [7, 29]. It is needed to increase risk perception among Chinese MSM.

Several previous studies found that MSM may be even less likely to be tested for STDs than HIV [31–33]. We did not test for STDs besides syphilis, but 33% of our study participants reported a history of STDs. Participants with high risk perception had an odds of >2 times of reporting a history of STDs than those in other risk perception groups. Hence, the promotion of STD testing can also be used as a complementary strategy to enhance risk perception among MSM.

Sex with both men and women is common among Chinese MSM. Nearly 60% of participants with no perceived risk of HIV infection reported ever had sex with female sexual partners, higher than those having a medium level of risk perception (40%). The potential for transmitting HIV from bisexual men to either men or women results largely from less condom use and sex with other MSM and women [34, 35]. Risk perception education should emphasize safe sex with both male and female sex partners.

Over half of our study sample received college education and were employed full time. Overall, there was no statistically significant relationship between education or employment with self-perceived risk, but a number of participants with high education and full-time employment thought that they were unlikely to acquire HIV. The risk awareness education program should consider that (1) MSM with lower educational attainment needs the basic information about the growing HIV/AIDS epidemic among MSM in China and the mechanisms of HIV transmission; (2) those with a higher level of education need more in-depth information about the importance of knowing HIV status through HIV testing and information to dispel incorrect information about HIV risk, for example, (1) AIDS is an irrelevant disease; (2) some partners are less risky than others; and (3) cleaning after sex is one way to prevent from HIV transmission [19].

There are some limitations in our study. First, all questionnaire data were based on self-reporting; study participants may have provided responses based more on social desirability to please the interviewers than actual experiences, especially experiences involving sensitive questions. Therefore, it is unknown whether participants under-reported or over-reported their involvement in risky behaviors. Second, the potential factors for HIV risk perception may not be accurately measured using a single question. Third, the MSM who participated in this study are volunteers recruited using nonrandomized sampling. The study sample may not represent the entire MSM community in Beijing or in China. Finally, the potential reasons of low HIV risk perception were not investigated; qualitative assessment may explore these reasons.

## 5. Conclusions

In summary, this study found that few MSM have high self-perceived HIV risk, even with HIV at an epidemic level among MSM in Beijing, China. HIV prevention programs should emphasize increasing HIV risk awareness among Chinese MSM, particularly those who are unemployed and uneducated.

## Figures and Tables

**Table 1:** Basic characteristics of men who have sex with men (MSM) in Beijing, China.

Characteristics^a^	*n* (*N* = 887)	%
Age at interview (year): mean ± SD^b^ (range)	30.2 ± 7.9 (18–67)	
≤23	148	16.7
24–29	344	38.8
30–39	278	31.3
≥40	87	13.2
Ethnicity		
Han	834	94.0
Others	53	6.0
Ever married		
Never	647	72.9
Ever	240	27.1
Education (year)		
Primary school and lower (≤6)	23	2.6
Middle school (7–9)	145	16.4
Senior high (10–12)	253	28.5
College and higher (>12)	465	52.5
Employment status		
Full-time/tenured	565	63.9
Part-time/temporary	186	21.1
Retired/unemployed	47	5.3
Student	46	5.2
Others	40	4.5
Resident in Beijing		
No	691	78.0
Yes	195	22.0
Illicit drug use		
Never	861	97.2
Ever	25	2.8
Alcohol drinking in the past 4 weeks		
Never	41	4.6
Rarely	176	19.9
2–6 times per week	449	50.7
Daily	220	24.8
Sexual orientation		
Homosexual	577	65.7
Heterosexual	10	1.2
Bisexual	291	33.1
Preferred sexual position during anal sex		
Exclusively receptive	103	11.6
Mainly receptive	155	17.5
Exclusively insertive	181	20.4
Mainly insertive	224	25.2
Dual	193	21.8
No anal sex	31	3.5
Self-perceived risk of HIV infection		
High	65	7.3
Medium	248	28.0
Low	463	52.2
No	111	12.5
HIV testing times since last HIV testing (month)		
Never	30	3.4
<3	282	31.8
3–5	326	36.8
6–11	132	14.9
≥12	117	13.2
HIV seropositive		
No	840	94.7
Yes	47	5.3
Syphilis seropositive		
No	739	83.3
Yes	148	16.7

^a^Sample size may vary for different characteristic variables due to missing data.

^b^SD: standard deviation.

**Table 2:** Univariate analysis of factors associated with self-perceived risk of HIV infection among MSM in Beijing, China.

Factors^a^	Self-perceived HIV risk % (*N*)				Crude odds ratio (95% confidence interval)		
	High risk (*n* = 65)	Medium risk (*n* = 248)	Low risk (*n* = 463)	No risk (*n* = 111)	High versus medium	Low versus medium	No versus medium
Age at interview (year):							
Mean ± standard deviation	28.1 ± 5.6	29.9 ± 7.2	30.6 ± 8.1	30.7 ± 9.2	0.96 (0.92, 1.00)	1.01 (0.99, 1.03)	1.01 (0.99, 1.04)
Ethnicity							
Han	89.2 (58)	96.0 (238)	93.1 (431)	96.4 (107)	1.00	1.00	1.00
Others	10.8 (7)	4.0 (10)	6.9 (32)	3.6 (4)	2.87 (1.05, 7.87)	1.77 (0.85, 3.66)	0.89 (0.27, 2.90)
Ever married							
Never	76.9 (50)	73.8 (183)	73.9 (342)	64.9 (72)	1.00	1.00	1.00
Ever	23.1 (15)	26.2 (65)	26.1 (121)	35.1 (39)	0.85 (0.44, 1.61)	1.00 (0.70, 1.42)	1.53 (0.94, 2.47)
Years of education							
≤12 years	40.0 (26)	50.8 (126)	43.0 (199)	64.0 (71)	1.00	1.00	1.00
>12 years	60.0 (39)	49.2 (122)	57.0 (264)	36.0 (40)	1.55 (0.89, 2.70)	1.37 (1.01, 1.87)	0.58 (0.37, 0.92)
Full-time employment							
No	35.4 (23)	43.3 (107)	31.8 (147)	38.2 (42)	1.00	1.00	1.00
Yes	64.6 (42)	56.7 (140)	38.2 (315)	61.8 (68)	1.40 (0.79, 2.46)	1.64 (1.19, 2.25)	1.24 (0.78, 1.96)
Residents in Beijing							
No	72.3 (47)	78.2 (194)	77.5 (359)	82.7 (91)	1.00	1.00	1.00
Yes	27.7 (18)	21.8 (54)	22.5 (104)	17.3 (19)	1.38 (0.74, 2.56)	1.04 (0.72, 1.51)	0.75 (0.42, 1.34)
Illicit drug use							
Never	95.4 (62)	95.1 (235)	98.7 (457)	96.4 (107)	1.00	1.00	1.00
Ever	4.6 (3)	4.9 (12)	1.3 (6)	3.6 (4)	0.95 (0.26, 3.46)	0.26 (0.10, 0.69)	0.73 (0.23, 2.32)
Alcohol drinking in the past 4 weeks							
Never	78.4 (51)	77.4 (192)	75.6 (350)	69.1 (76)	1.00	1.00	1.00
Rarely	13.9 (9)	19.0 (47)	20.3 (94)	23.6 (26)	0.72 (0.33, 1.57)	1.10 (0.74, 1.62)	1.40 (0.81, 2.42)
Often (≥2 times per week)	7.7 (5)	3.6 (9)	4.1 (19)	7.3 (8)	2.09 (0.67, 6.51)	1.16 (0.51, 2.61)	2.25 (0.84, 6.04)
Sexual orientation							
Homosexual	63.1 (41)	70.0 (170)	66.0 (305)	56.5 (61)	1.00	1.00	1.00
Heterosexual	1.5 (1)	1.2 (3)	0.9 (4)	1.8 (2)	1.38 (0.14, 13.63)	0.74 (0.16, 3.36)	1.86 (0.30, 11.39)
Bisexual	35.4 (23)	28.8 (70)	33.1 (153)	41.7 (45)	1.36 (0.76, 2.44)	1.22 (0.87, 1.71)	1.79 (1.11, 2.88)
Preferred anal position							
Exclusively/mainly receptive	33.9 (22)	32.5 (78)	29.0 (130)	27.2 (28)	1.00	1.00	1.00
Both insertive and receptive	21.5 (14)	23.8 (57)	23.0 (103)	18.5 (19)	0.87 (0.41, 1.85)	1.08 (0.71, 1.66)	0.93 (0.47, 1.82)
Exclusively/mainly insertive	44.6 (29)	43.8 (105)	48.0 (215)	54.4 (56)	0.98 (0.52, 1.83)	1.23 (0.85, 1.77)	1.49 (0.87, 2.55)
Unprotected anal sex in the past 6 months							
No	63.1 (41)	73.8 (183)	77.3 (358)	80.2 (89)	1.00	1.00	1.00
Yes	36.9 (24)	26.2 (65)	22.7 (105)	19.8 (22)	1.65 (0.93, 2.94)	0.83 (0.58, 1.18)	0.70 (0.40, 1.20)
Multiple concurrent male sexual partners in the past 12 months							
No	87.7 (57)	91.9 (228)	93.7 (434)	95.5 (106)	1.00	1.00	1.00
Yes	12.3 (8)	8.1 (20)	6.3 (29)	4.5 (5)	1.60 (0.67, 3.82)	0.76 (0.42, 1.38)	0.54 (0.20, 1.47)
Traded sex for money in the past 12 months							
No	92.3 (60)	95.2 (236)	96.5 (447)	91.9 (102)	1.00	1.00	1.00
Yes	7.7 (5)	4.8 (12)	3.5 (16)	8.1 (9)	1.64 (0.56, 4.83)	0.70 (0.33, 1.51)	1.74 (0.71, 4.25)
Forced to have sex with any male sexual partners in the past 12 months							
No	93.8 (61)	96.0 (238)	97.4 (451)	96.4 (107)	1.00	1.00	1.00
Yes	6.2 (4)	4.0 (10)	2.6 (12)	3.6 (4)	1.56 (0.47, 5.15)	0.63 (0.27, 1.49)	0.89 (0.27, 2.90)
Self-reported history of sexually transmitted diseases							
No	59.4 (38)	76.0 (184)	77.1 (346)	84.1 (90)	1.00	1.00	1.00
Yes	40.6 (26)	24.0 (58)	22.9 (103)	15.9 (17)	2.17 (1.22, 3.88)	0.94 (0.65, 1.36)	0.60 (0.33, 1.09)
Ever had sex with female sexual partner(s)							
No	52.3 (34)	59.7 (148)	55.3 (256)	42.3 (47)	1.00	1.00	1.00
Yes	47.7 (31)	40.3 (100)	44.7 (207)	57.7 (64)	1.35 (0.78, 2.34)	1.20 (0.88, 1.64)	2.02 (1.28, 3.17)
Circumcision status by exam							
Uncircumcised	92.2 (59)	90.3 (215)	92.6 (411)	92.7 (102)	1.00	1.00	1.00
Circumcised	7.8 (5)	9.7 (23)	7.4 (33)	7.3 (8)	0.79 (0.29, 2.17)	0.75 (0.43, 1.31)	0.73 (0.32, 1.70)
Time since last HIV testing (month)							
<3	44.6 (29)	27.8 (69)	30.9 (143)	36.9 (41)	1.00	1.00	1.00
≥3 or never	55.4 (36)	72.2 (179)	69.1 (320)	63.1 (70)	0.48 (0.27, 0.84)	0.86 (0.61, 1.21)	0.66 (0.41, 1.06)
HIV seropositivity							
No	89.2 (58)	93.2 (231)	96.3 (446)	94.6 (105)	1.00	1.00	1.00
Yes	10.8 (7)	6.8 (17)	3.7 (17)	5.4 (6)	1.64 (0.65, 4.14)	0.52 (0.26, 1.03)	0.78 (0.30, 2.03)
Syphilis seropositivity							
No	78.5 (51)	81.0 (201)	86.2 (399)	79.3 (88)	1.00	1.00	1.00
Yes	21.5 (14)	19.0 (47)	13.8 (64)	20.7 (23)	1.17 (0.60, 2.30)	0.69 (0.45, 1.04)	1.12 (0.64, 1.95)

^a^Sample size may vary for different characteristic variables due to missing data.

**Table 3:** Multivariate analysis of factors associated with self-perceived risk of HIV infection among MSM in Beijing, China.

Factors	Adjusted OR (95% CI)		
	High versus medium	Low versus medium	No versus medium
Ethnicity			
Others versus Han	2.91 (1.03, 8.19)	—	—
Full-time employment			
Yes versus no	—	1.58 (1.15, 2.18)	—
Illicit drug use			
Ever versus never	—	0.28 (0.10, 0.75)	—
History of sexually transmitted diseases			
Yes versus no	2.27 (1.25, 4.10)	—	—
Ever had sex with female sexual partner(s)			
Yes versus no	—	—	1.93 (1.22, 3.05)
HIV testing times since last HIV testing (months)			
≥3 or never versus <3	0.47 (0.26, 0.84)	—	—

Note: OR: odds ratio; CI: confidence interval.

**Table 4:** Association of HIV risk perception with HIV and syphilis infections among MSM in Beijing, China.

Self-perceived risk of HIV infection	Unadjusted OR (95% CI)	*P* value	Adjusted OR* (95% CI)	*P* value
HIV seropositivity				
High risk	Ref.		Ref.	
Medium risk	0.61 (0.24, 1.54)	0.29	0.60 (0.23, 1.54)	0.28
Low risk	0.32 (0.13, 0.79)	0.01	0.35 (0.14, 0.90)	0.03
No risk	0.47 (0.15, 1.48)	0.20	0.46 (0.15, 1.49)	0.20
*P*_trend_		*0.07 *		*0.16 *
Syphilis seropositivity				
High risk	Ref.		Ref.	
Medium risk	0.85 (0.44, 1.67)	0.64	0.77 (0.39, 1.53)	0.46
Low risk	0.58 (0.31, 1.12)	0.10	0.53 (0.27, 1.02)	0.06
No risk	0.95 (0.45, 2.01)	0.90	0.78 (0.36, 1.68)	0.53
*P*_trend_		*0.11 *		*0.12 *

Note: OR: odds ratio; CI: confidence interval.

*Adjusted for age, ethnic, years of education, and full-time employment.
